# Simulation of the sensing mechanism in quantum dot gas sensor by quantum light harvesting approach

**DOI:** 10.3389/fchem.2022.1036197

**Published:** 2022-10-17

**Authors:** Ongart Suntijitrungruang, Jidapa Lakronwat, Teerapat Uthailiang, Peera Pongkitiwanichakul, S. Boonchui

**Affiliations:** ^1^ Department of Physics, Faculty of Science, Kasetsart University, Bangkok, Thailand; ^2^ Center of Rubber and Polymer Materials in Agriculture and Industry (RPM), Faculty of Science, Kasetsart University, Bangkok, Thailand

**Keywords:** quantum dot gas sensor, quantum light harvesting, energy transfer efficiency, Schottky barrier, phonon baths

## Abstract

Quantum dot (QD) gas sensors are one of the most useful nanotechnologies applied to protect people from unnecessary harm. This work theoretically explores the mechanism in QD gas sensors in order to advance the prudent design of relevant products. The theoretical model employed in this research is similar to the process in plants’ photosynthesis, referred to as charge separation of light harvesting. In this work, we investigate the details of energy transport in QD gas sensors carried by electrons from the circuit. We demonstrate theoretically how the effects of temperature and gas detection affect electron transport. To analyze thoroughly, the potential energy referred to as the Schotthy barrier perturbed by gasses is considered. Moreover, the energy transfer efficiency (ETE) of QD gas sensors for oxidizing or reducing gas is shown in the simulation. The results imply that the electron transport between QDs (raising the current and lessening the current) depends on a parameter corresponding with the Schotthy barrier. In regard to thermal energy portrayed by phonon baths, a higher temperature shortens the time duration of energy transport in QDs, hence raising energy transfer efficiency and energy current. Our model can be applied to further QD gas sensors’ design and manufacture.

## 1 Introduction

In 2017, air pollution was a serious problem, causing ∼4.9 million premature deaths (Zhang et al., 2021). Therefore, it is necessary to advance the gas sensors technology for protecting people from deadly toxic harm. Currently, there are various types of gas detectors, such as electrochemical (Pletcher et al., 1991; Naoyoshi, 1972), ultrasonic (Westerveld Wouter, 1993), and semiconductor (Jianhai et al., 2018; Murali et al., 2020). Quantum dots (QDs), also called artificial atoms (Fox, 2010), are semiconductor particles a few nanometers in size, possessing unique electronic and optical properties due to quantum mechanics (Thitapura et al., 2017; Fanbanrai et al., 2015). QDs, presently, are one of the most promising nanotechnologies utilized and studied in diverse ways. For instance, Srisangyingcharoen et al. (2015) investigated the dynamics of photon emission from QDs that induce surface plasmon propagation. Leschkies et al. (2007) demonstrated how ZnO nanowires with CdSe QDs can improve solar cells to 50–60% internal quantum efficiencies. Through extraordinary properties, QDs can be integrated with gas sensors for certain benefits. Zhilong et al. (2018) fabricated a fully stretchable and humidity-resistant gas sensor by quantum dots. This enables the usage of a wide variety of substrates and offers many degrees of freedom in sensor design (Saran and Curry, 2016). Mosadegh Sedghi et al. (2010), due to good chemical stability and functional properties of metal oxide (Galstyan et al., 2013; Galstyan, 2017; Lzhijie et al., 2019), compared the sensing response of SnO_2_ QDs to the conventional SnO_2_ sensors; this was one of their pioneering works. Also, the metal chalcogenide QDs (such as PbS QDs), after the treatment using sodium nitrite (NaNO_2_), demonstrate a very high response towards 50 ppm of NO_2_ (Liu et al., 2014). Owing to these promising characteristics, it is significant to theoretically investigate the mechanism of QD gas sensors in order to discover ways for developing relevant devices.

The energy transport in QD gas sensors is generally impacted by the coupling Hamiltonian between QDs in common with the hopping Hamiltonian of chlorophyll’s pigments in photosynthesis systems. Normally, when QD sensors detect particular gasses, they perturb the Hamiltonian of the sensor’s system, resulting in the response. Theoretically, such a response involves altered energy transport in nanoside sensors. To study the characteristics of the perturbed Hamiltonian in QD sensors, the transport model is very appropriate. This feature is suitable for analyzing coupled molecular systems, such as photosynthetic complexes (Caruso et al., 2009; Wang et al., 2018; Tomassi and Ivan, 2020) and organic photovoltaic cells (Yonatan and Ventra, 2011). Moreover, the number of studies emphasizing the quantum description of transport has sharply increased (Rebentrost et al., 2009; Jang et al., 2014; Maier et al., 2019; Moreira et al., 2020; Pei-Yun and Cao, 2021), (Charoenpakdee et al., 2020). The energy transport model, as well, can be employed for investigating quantum invasiveness (Moreira and Cunha, 2019) and coherence (Baumgratz et al., 2014). Furthermore, through this approach, portraying the bath effect on the system, it is proper to articulate the transport model with the QD gas sensor’s system. Sklinkla et al. (2015) exemplified how the charge perturbs the certain field in a nanosphere pair. We explore and report two different kinds of perturbed systems. We additionally investigate how temperature affects the system by studying the phonon interaction with the QDs. Finally, we describe the conclusion of our theoretical QD gas sensor model, which is similar to the charge separation and light harvesting in photosynthesis complexes.

For the gas-surface of semiconductor interaction, there is gas absorption on the surface of semiconductors. Our work theoretically demonstrates the QD gas sensor system based on the quantum energy transport model. Also, we show how particular gasses impact the QD system as the perturbation that alters the electrical properties in the sensor, generating the signal. Ordinarily, the main component of air is oxygen. Therefore, the interaction of semiconductor materials with oxygen plays a crucial role in identifying the presence of other gasses in the environment. In addition, the interaction between the gasses and the surface is called redox which may reduce or increase the ionosorption of oxygen on QDs surface. Depending on the type, gasses can donate or accept electrons in the system. Thus, there are two characteristics in a redox interaction, leading to two different perturbations in the system. The first is gasses reducing to the sensor, that is, the sensor receives electrons from the gasses. The other case is the sensor losing electrons to the gasses, implying that certain gasses are electron acceptors.

## 2 Charge transport model

**FIGURE 1:**
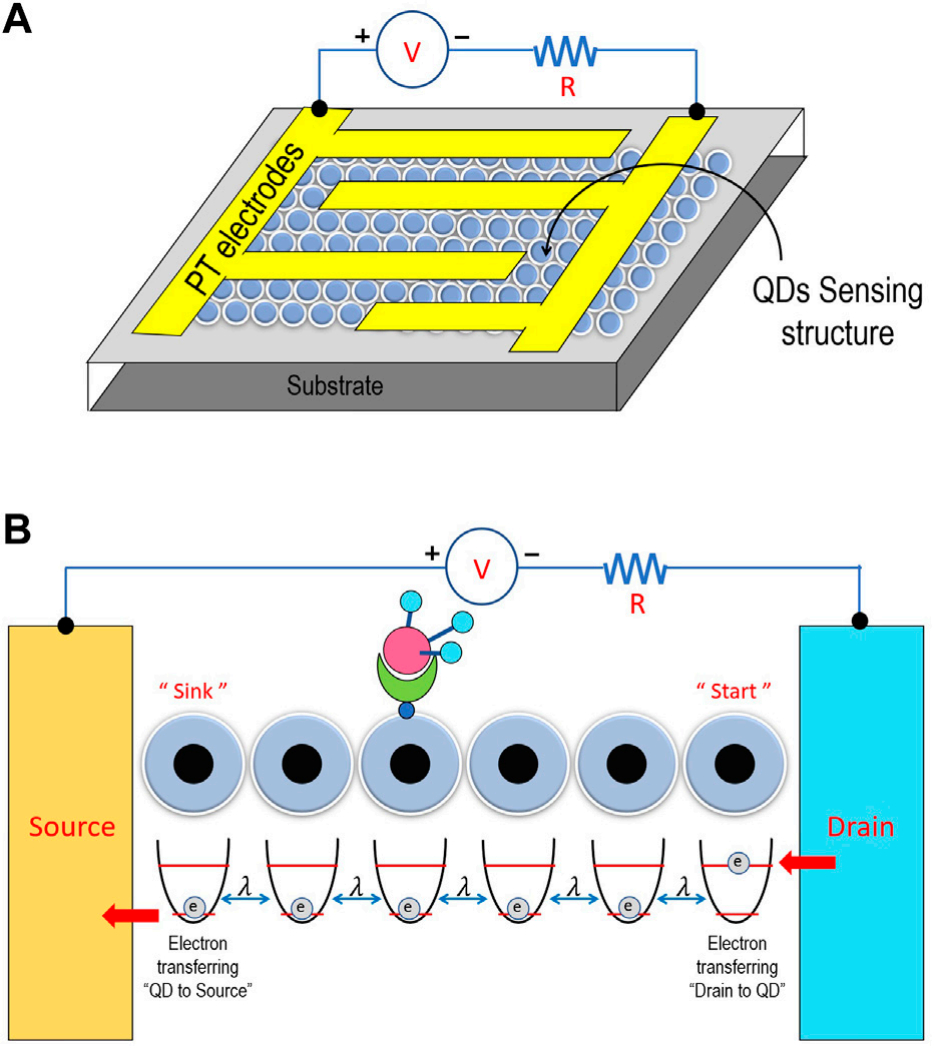
**(A)** Schematic of a chemiresistive structure with platinum (Pt) electrodes and Pt heater for gas sensing measurements. **(B)** Schematic diagram of a linear chain of *N*-coupled two-level systems (quantum dots (QDs)); that is, the QDs integrated into the sensing structures have been mainly synthesized by colloidal methods. The QD 4^th^ site is surfaced by using a functional group, which is used to detect the analyte.

In photosynthesis, energy (or exciton) is transported from the initial chlorophyll’s pigment to the final pigment called the reaction center. The energy transport in charge separation involves the thermal energy modeled by phonon heat baths. In the same manner, electron transport in QD gas sensors is transferred from the initial QD to the final QD, which entails photon heat baths of each QD. Therefore, in this work, where we investigate the energy transport in QD gas sensors, we utilize the model resembling the exciton transport in the photosynthesis process. Using our selected model, this work took into account a transport condition. A linear chain of *N*-coupled two-level systems describes it (sites), as shown in Figure 1. The parameter determines the degree of connection between the locations. In addition, each site is exposed to a local environment that, at rates, induces dephasing and spontaneous emission *γ* and Γ_0_, respectively. The final location is irrationally connected to a sink where the energy is collected. Energy transport, as previously indicated, is a key component of coupled molecular systems, including photosynthetic complexes (Wang et al., 2018) and organic photovoltaic cells(Yonatan and Ventra, 2011). The model shown in Figure 1B, which consists of a linear chain of *N* first-neighbor connected-level systems, captures its key characteristics (sites).

The system Hamiltonian ${\hat{H}}_{0}={\hat{H}}_{QD}+{\hat{H}}_{I}$ consists of a free part and a part which accounts for the coupling between first neighbors in the chain

$${\hat{H}}_{QD}=\overset{N}{\underset{j=1}{\sum }}\hbar \omega_{j}\sigma_{z}^{\left(j\right)},\;\;\;\text{and }\;\;\;{\hat{H}}_{I}=\overset{N-1}{\underset{j=1}{\sum }}\hbar \lambda_{j,j+1}\left(\sigma_{+}^{\left(j\right)}\sigma_{-}^{\left(j+1\right)}+\sigma_{-}^{\left(j\right)}\sigma_{+}^{\left(j+1\right)}\right);\;\;j\;\;\text{is the site index}.$$ (1)

**FIGURE 2:**
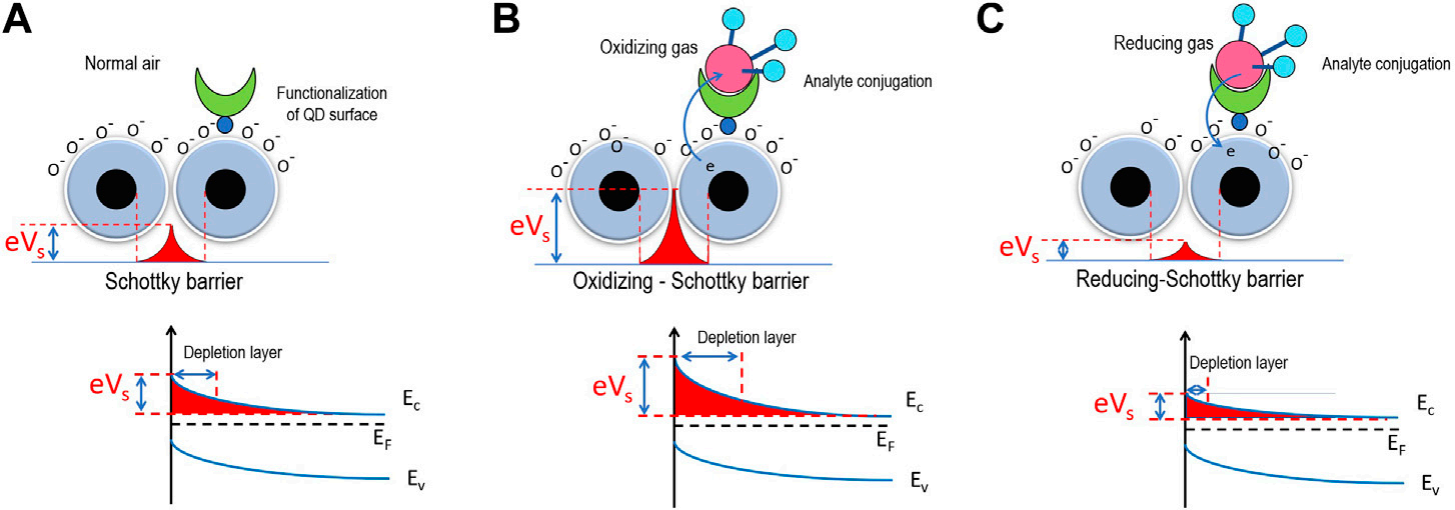
Schematic mechanism of gas detection which changes the space charge layer or Schottky barrier. **(A)** Normal air, **(B)** oxidizing gas (*λ*_*j*,*j*+1_ reducing), and **(C)** reducing gas (*λ*_*j*,*j*+1_ increasing).

At lower temperatures, O_2_ is absorbed while $O_{2}^{-}$ can alter to be O^−^ at higher temperatures. As a result, such processes of gas absorption and ion alteration depend on the temperature (Yan et al., 2020). The ionosorbed oxygen kinds extract the electrons from the conduction band of QD, leading to the formation of a depletion layer and a Schottky barrier at the grain boundaries, shown in Figure 2A. Generally, the principle of the QD gas sensor is the modulation of the electrical characteristic. Based on attributes of the sensing layer and the nature of the gas, the potential energy of the Schottky barrier between QDs changes by corresponding to the types of gas. For example, in particular materials such as SnO_2_ (QD particles), an electron depletion layer exists originally in the air by adsorbed oxygen ions and subsequent uncovering to a reducing gas. Then, the liberated electrons return to the surface of the sensing layer, narrowing the width of the electron depletion layer. Eventually, these contribute to the sensor signal. Considering the perturbed Schottky barrier between QDs after the sensor detects the gasses, this perturbation tendency depends on the redox interaction type of gasses referred to as oxidation and reduction interaction. In our model, the effects of the Schottky barrier between neighboring QDs the *j* th and the *j* + 1 th, such as depletion layer or height of potential, represents by a coupling parameter *λ*_*j*,*j*+1_. As a result of gas detection effects, the coupling parameter is changed, leading to altered charge transfer flow in our model. Similar to the photosynthesis process, the coupling energy between each pigment in chlorophyll determines the strength of the charge transfer. Physically, the coupling energy originate from the F $\ddot{o}$ rster dipole–dipole interaction. In photosynthesis, the dipole interaction expedites charge separation due to quantum light harvesting. This is a motivation in our calculation. For an electron transfer between QDs, the electron has to transport through the Schottky barrier. There is an intimate contact that provides feasible charge transport. If the energy of the electron is higher than the height of the Schottky barrier, the electron can transmit across the Schottky barrier. On the other hand, if the energy of the electron is less than the height of the Schottky barrier, the electron incident from one side can appear on the opposite side of this barrier by the phenomenon known as barrier penetration or tunneling.

In addition, if the sensors detect oxidizing gasses (such as NO, NO_2_, and O_3_) that receive electrons from QDs, the Schottky barrier increases, as shown in Figure 2B. This effect causes a high Schottky barrier and high depletion layer that hinders electron transport. On the contrary, if the sensors detect reducing gasses (such as CO, NH_3_, and C_2_H_5_OH) that release electrons to QDs, the Schottky barrier and depletion layer are reduced, hence accelerating electron transport. Therefore, we can investigate the effect from two different gas types by altering the parameter *λ*_*j*,*j*+1_. The energy *ℏω* is associated with each two-level system, whereas ***σ***_±_ and ***σ***_*z*_ are the Pauli matrices,

$$\sigma_{-}=\left(\begin{array}{cc} 0 & 1\\ 0 & 0 \end{array}\right),\sigma_{+}=\left(\begin{array}{cc} 0 & 0\\ 1 & 0 \end{array}\right),\sigma_{z}=\left(\begin{array}{cc} 1 & 0\\ 0 & -1 \end{array}\right).$$ (2)

By adding one excitation to the chain’s first site and none to the others, the system is brought back to its starting state. We assume that the *N* th site of the chain is dissipating into an additional two-level system known as the sink *s* in order to assess the transport efficiency. In order to account for the existence of noise, we also take into account that each site in the chain is subject to local dissipation and local dephasing. We observe that the outcomes shown below appear to be independent of the regional frequencies. Physically speaking, this mechanism is equivalent to nearest neighbor energy migration that is thermally initiated (Vaziri and Plenio, 2010). Consequently, the master equation yields the dynamics of the basic transport case,

$$\frac{\partial \hat{\rho }}{\partial t}=-\frac{i}{\hbar }\left[\hat{\rho },{\hat{H}}_{0}\right]+L_{\mathrm{sink}}\hat{\rho }+\overset{N}{\underset{j=1}{\sum }}L_{j}\hat{\rho },$$ (3)

where $\hat{\rho }$ is a density matrix which discusses the phenomenon of energy transport in the chain of *N*-coupled two-level systems. In this scenario, the noise in each site *j* is described by the Lindblad superoperator,

$${\hat{L}}_{j}\hat{\rho }=\Gamma_{0}\left(2\sigma_{+}^{\left(j\right)}\hat{\rho }\sigma_{-}^{\left(j\right)}-\sigma_{-}^{\left(j\right)}\sigma_{+}^{\left(j\right)}\hat{\rho }-\hat{\rho }\sigma_{-}^{\left(j\right)}\sigma_{+}^{\left(j\right)}\right)+\left.\gamma \left(\sigma_{z}^{\left(j\right)}\hat{\rho }\sigma_{z}^{\left(j\right)}-\hat{\rho }\right)\right).$$ (4)

For an example case (Ohmic case (Haikka et al., 2013)), the dissipation rates Γ_0_ is written as

$$\Gamma_{0}=2k_{B}T\Gamma \left[s-1\right]\sin\left[\left(s-1\right)\text{arctan}\left(\omega_{c}t\right)\right],$$ (5)

and Γ[*s*] is the Euler gamma function, *ω*_*c*_ is the reservoir cutoff frequency, and *γ* is the dephasing rates which are the spontaneous decay rates of the exciton state of the *m* th QD that can be derived from Fermi’s golden rule. In turn, the coupling between the *N* th site and the sink is described by

$${\hat{L}}_{\mathrm{sink}}\hat{\rho }=\Gamma_{s}\left(2\sigma_{-}^{\left(N\right)}\sigma_{+}^{\left(s\right)}\hat{\rho }\sigma_{-}^{\left(s\right)}\sigma_{+}^{\left(N\right)}-\sigma_{-}^{\left(s\right)}\sigma_{+}^{\left(N\right)}\sigma_{-}^{\left(N\right)}\sigma_{-}^{\left(s\right)}\hat{\rho }-\hat{\rho }\sigma_{-}^{\left(s\right)}\sigma_{+}^{\left(N\right)}\sigma_{-}^{\left(N\right)}\sigma_{-}^{\left(s\right)}\right),$$ (6)

where Γ_*s*_ > 0 is the rate of energy transferred to the sink. The integrated probability of the excitation successfully exiting the channel and reaching the acceptor is known as the energy transfer efficiency (ETE) of the channel. Following Mohseni et al. (2008)’s work, ETE *η* is defined as

$$\eta =\frac{1}{T}\int_{0}^{T}\mathrm{Tr}\left(|e_{s}\rangle \langle e_{s}|\hat{\rho }\left(t\right)\right)dt.$$ (7)

This definition, using integrated success probabilities, was used in the context of energy transfer from donor to acceptors. An energy current operator can be derived for a general multi-site Hamiltonian (Medina et al., 2013). The expression for an energy current operator $\vec{j}\left(x\right)$ can be obtained from a continuity equation

$$\frac{d}{dt}H\left(t\right)+\vec{\nabla }\cdot \vec{j}\left(x\right)=0,$$ (8)

where $H\left(t\right)=\mathrm{Tr}\left({\hat{H}}_{QD}\hat{\rho }\left(t\right)\right)$ is the local energy density and identifies $\vec{j}\left(x\right)$ the energy current as

$$\vec{j}\left(x\right)=\mathrm{Tr}\left(\hat{\vec{j}}\hat{\rho }\left(t\right)\right)\;\;\;\;\text{and}\;\;\;\;\hat{\vec{j}}={\hat{s}}_{i\to s}-{\hat{s}}_{s\to i},$$ (9)

and the directed energy current from the site *i* to the sink *s* is defined as ${\hat{s}}_{i\to s}=|e_{s}\rangle \langle e_{i}|$ .

## 3 Numerical results

### 3.1 Temperature effects on energy transfer in a linear chain of QDs in the gas sensor

**FIGURE 3:**
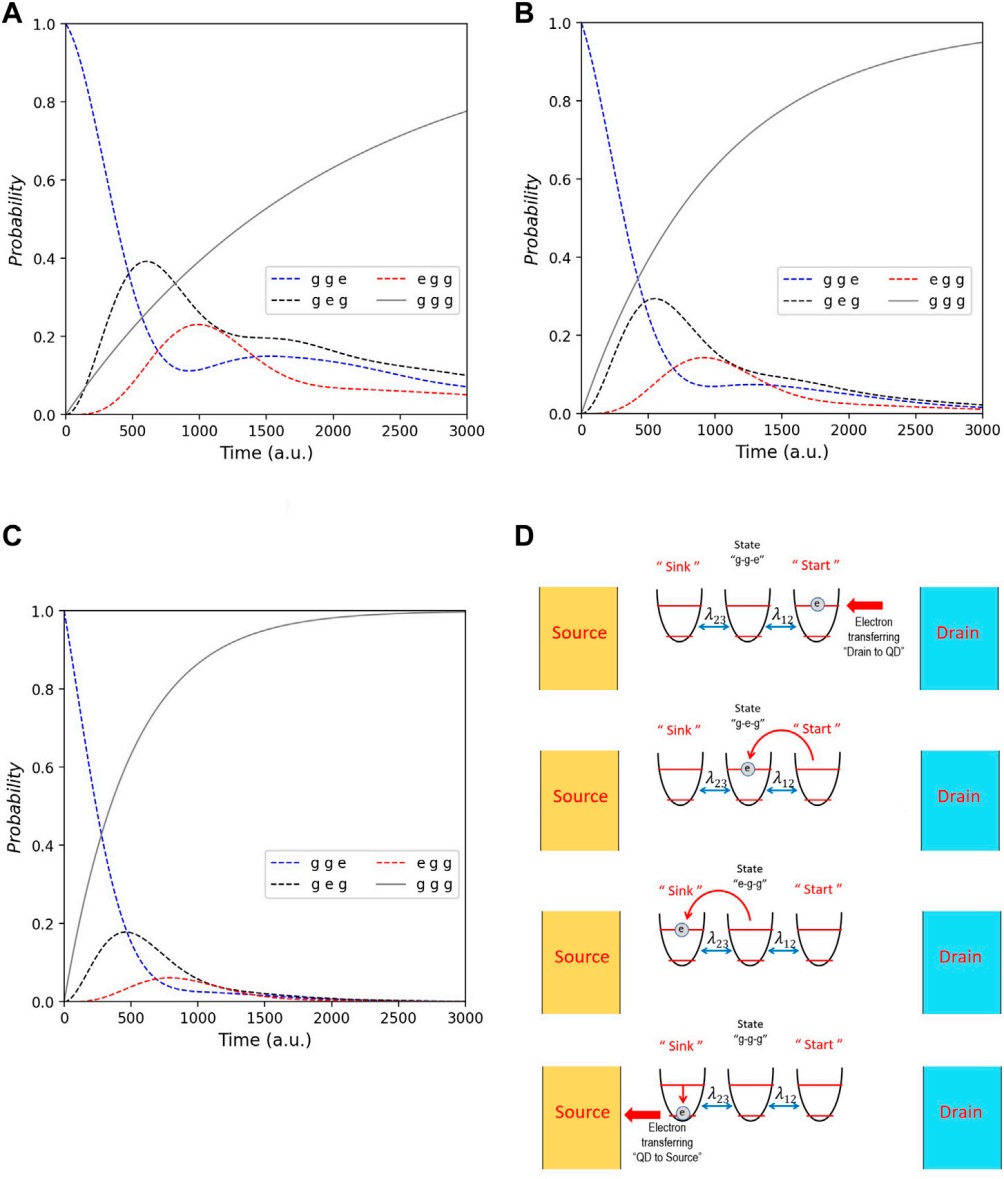
Probability of all states that are *gge* (blue line), *geg* (black line), *egg* (red line), and *ggg* (gray line) for *λ*_1,2_ = 2 (a.u.). Each picture demonstrates the whole tendency of probability with different Γ_0_ that are 0.25 for **(A)**, 0.5 for **(B),** and 1 for **(C)**. **(D)** Schematic mechanism of energy transport beginning from the drain gate to the source gate.

In our model, the phonon baths entail the temperature corresponding to the parameter Γ_0_ in common with the works (Mohseni et al., 2008; Haikka et al., 2013). First, we explore the QD gas sensor system without gas. QD sensors usually consist of the gas detector dot, sink dot and other dots. Therefore, the QD system in gas sensors can be simplified into three dots. As shown in Figure 3D, the first dot receives an electron from the drain gate of the circuit. Then, the electron transfers to the next dot and eventually to the sink dot. After the electron transports to the sink dot, the source gate will gain this electron. Ultimately, all dots will be ground state.

Figure 3 shows the dynamics of QDs states according to QDs in Figure 2A that are without any gas. Figures 3A, B, and C show the result of all states from the different Γ_0_ = 0.25, 0, 5, and 1 (a.u.), respectively. In our simulation, we determine the sink is the third dot, and the initial state is *gge* (that is, the electron in the first dot only is excited). Hence, the probability of state *g*, *g*, *e* is highest at *t* = 0 in every graph. However, as time goes on, the initial state decays continuously and monotonically and becomes zero at the end. Comparing Figures 3A, B, and C, it is obvious that the time duration starting from initial to final (where nearly all dots are ground state) depends on Γ_0_. Theoretically, Γ_0_ corresponds to the phonon bath related to the temperature. That is, if the temperature increases, the energy transport will be swifter. We, on this ground, can modify gas sensors through temperature control. The experimental work by Galstyan (2021) shows how the temperature affects the response of QD sensors, conforming to our result. Additionally, while the time duration (for energy transport in each case) is different, the probability’s tendency of each temperature is the same.

Considering the whole tendency of Figure 3, the state’s probability (*ggg*) is zero at the starting point but is one at the final, hence implying the energy of QDs has decreased. Such results illustrate that the energy in the system is transferred to the source gate where electrons transport to ultimately. Analyzing the probability of intermediate states (*geg* and *egg*), these are nonmonotonic that increase at their first phase and then decay. The simulation shows that the state *geg* grows and decays before the state *egg*. It means that electrons from the first dot move to the second dot and then to the sink dot. Eventually, after the dots lost their energy to the source gate consistently, the probability of intermediate state decay to be the final state *ggg*. Concerning the peak of the intermediate state’s probability, we find that more Γ_0_ (that is, the more temperature) makes the peak decrease. This means that more temperature induces electrons to move into the source gate more swiftly, as concluded from the decreased probability of intermediate states.

**FIGURE 4:**
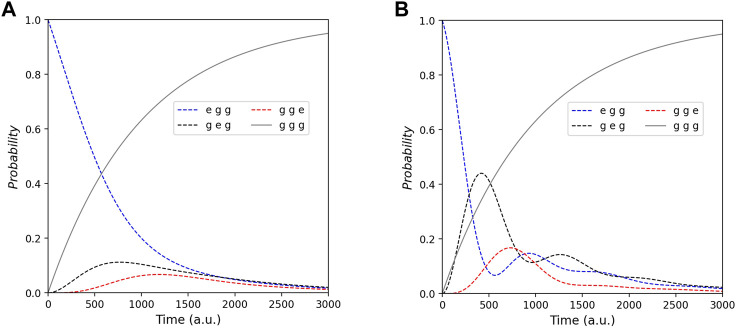
Probability of all states that are *gge* (blue line), *geg* (black line), *egg* (red line), and *ggg* (gray line) at Γ_0_ = 0.5 (a.u.). Each picture demonstrates the whole tendency of probability with different *λ*_1,2_ that are **(A)** 1 and **(B)** 3 (a.u.).

Regarding ETE, as we define in Eq. 8, we can observe roughly by considering the growth of the final state *ggg*. As shown in Figure 4, we discover that more Γ_0_ make the final state grow rapidly. Since this parameter directly relates to the temperature, our results demonstrate that the more the temperature, the more the ETE, thereby harmonizing with the experimental works by Rajneesh Kumar et al. (2021), which shows the ZnS QDs sensor’s acetone sensing properties for various concentrations (20−100 *ppm*) at different operating temperatures (100−200°C). It showed that the sensitivity of the ZnS QDs sensor increases with operating temperature and gas concentration. Thoroughly, by Eq. 8, ETE in each case is 0.482, 0.683, and 0.834 for Γ_0_ = 0.25, 0.5, and 1 (a.u.), respectively. On this ground, our work theoretically suggests a way for improving and modifying the QD sensors by controlling temperature.

### 3.2 Analyzing probability and energy current from gas detection effects with the Schottky barrier in the QD gas sensor

Normally when the system detects gasses, the energy potential referred to as the Schottky barrier between QDs is perturbed. Due to gas detection, the coupling energy between QDs is perturbed, according to the experimental research by Chowdhurya and Bhowmik (2021). This perturbation corresponds to the parameter *λ*_*j*,*j*+1_ in Eq. 1. Since we simplify the QD gas sensors to be three dots, the effects of gas detection impact the coupling energy between the first and the second (the detection) dot, that is *λ*_1,2_, whereas *λ*_2,3_ is invariable. We, thus, can investigate the result of gas detection in the QD system by varying this parameter in simulations. Figure 4 illustrates the effect after the system detects gasses. In our simulation, we determine the second QD is the detector dot. Generally, the effect of gas detection may increase or decrease the Schottky barrier depending on the type of gas. In order to study this effect prudently, we simulate in both cases (increasing and decreasing the Schottky barrier). Figure 4A is the case of the increased Schottky barrier where the coupling parameter (*λ*_1,2_) decreases. On the other hand, Figure 4B is the simulation from increased *λ*_1,2_, that is, the case of the decreased Schottky barrier.

Comparing Figure 3B with Figure 4A and Figure 4B, the parameters in these simulations are the same (except *λ*_1,2_). In this regard, we employ Figure 3B as the system without gas where there is no perturbation. We notice the final state (*ggg*) is the same in these simulations while the other states are altered. Thus, for manufacturing QD gas sensors, the response mechanism of the products should depend on the altered probability of these other states. Focusing on Figure 4B where the coupling parameter increases, it shows the strong oscillation between the state *geg* and *gge*. Also, there is the oscillation between *egg* and *geg*. This demonstrates that the increased coupling parameters make the electron transfer (between the first and second dot) easier. On the contrary, Figure 4A, where the coupling parameter decreases, it shows less oscillation between the first and second dot. Analyzing the intermediate states *geg* and *egg* (as shown in black and red lines), these states in Figure 4B grow more rapidly than in Figure 4A. That is, the strong coupling makes the electrons transport between QDs more comfortably. However, we see that the growth of the final state (*ggg*) is the same despite two different types of gasses. Accordingly, ETE does not rely on the gas type, whether oxidizing or reducing gas. Concerning the energy current that we can observe from the probability’s summation of the final state and intermediate states as defined in Eq. 9, Figure 4 demonstrates roughly that the more coupling energy, the more energy current since the increased *λ*_1,2_ causes the probability of intermediate states to increase.

**FIGURE 5:**
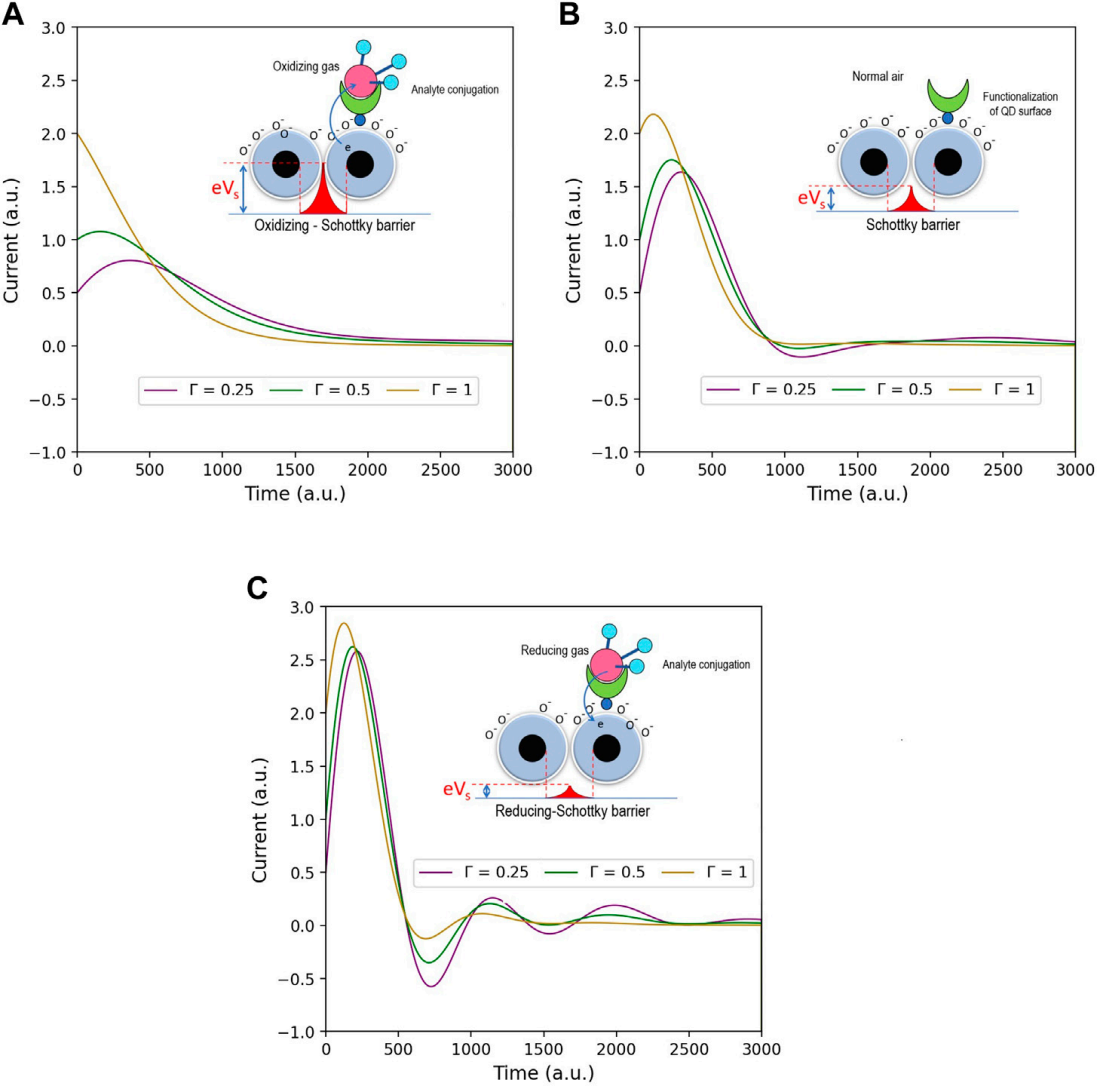
Show the energy current of QD gas sensors with Γ_0_ varied to be **(A)** 0.25, **(B)** 0.5, and **(C)** 1 (a.u.), whereas *λ*_1,2_ is invariable. Inset picture assumes three Schottky barrier cases **(A)** for increased Schottky barrier (reducing gas), *λ*_1,2_ = 1 (a.u.), **(B)** for normal Schottky barrier (normal gas), *λ*_1,2_ = 2 (a.u.), and finally, **(C)** for decreased Schottky barrier (oxidizing gas), *λ*_1,2_ = 3 (a.u.).

Energy current is one of the most essential parts of QDs sensors. We, therefore, investigate the characteristics of energy current involved in the temperature and gas detection effects. Figure 5 is the simulation focusing on the energy current with different coupling energy and temperature. As shown by the figures, Figure 5B shows the result from the undetected gas system, whereas Figure 5A and Figure 5C are from the system that detects gasses both in increased and decreased Schottky barrier. Comparing the case of the increased Schottky barrier that gasses accept electrons from QDs (Figure 5A) with the undetected gas system (Figure 5B), the simulation portrays vividly that the increased Schottky barrier (decreased coupling energy) leads the whole energy current lower. This implies that the accepter gasses impede electron transport in the structure. On the other hand, the decreased Schottky barrier (increased coupling energy, as shown in Figure 5C) causes the overall energy current to be higher. Accordingly, the donor gasses accelerate electron transport. As explained in the former topic, the stronger coupling energy makes electron transfer between QDs easier, thereby the more energy current.

Considering the effect of temperature corresponding to the parameter Γ_0_, we see that the higher temperature obviously raises the energy current. The thermal energy generally relates to the phonon baths. Accordingly, more interaction between phonon baths and QDs (depending on the temperature) expedites electron transport, hence increasing energy current. On this ground, to design QD gas sensors, manufacturers can create the products from the response of altered energy currents when the sensors detect particular gasses.

## 4 Discussion

We have developed a fundamental method, resembling charge separation in photosynthesis, to explain charge transfer in QD gas sensors by using master equations. Yet the results shed light on the roles played by information, charge density, and charge transfer in QDs in sensor devices and materials. According to our work, the electron transport in the QD gas sensor corresponds to the temperature and the perturbed Schottky barrier, as shown by our simulation. By this mechanism, the electrical properties in the system, such as resistance and current, are changed as a result of gas detection effects. For this reason, the essential working principle of QD gas sensors involves the altered electrical characteristics inside the devices. The QD gas sensor is one of the gas detection types that are metal-oxide-semiconductor sensors (MOS sensors). Generally, this type of sensor employs chemical reactions that take place when the specific gas comes in direct contact with the sensor for generating the detection signal. Furthermore, through the change of electrical attributes in the system, the sensor can analyze the gas concentration. However, our model is a simplified system that consists of three QDs. To investigate gas concentration further, our model can be improved by adding other QDs for detecting the gasses.

At present, there are various types of the gas sensors, such as electrochemical, catalytic bead, photoionization, infrared point, and ultrasonic sensors. Each type has pros and cons compared to others. For example, photoionization detectors, which use a high-photon-energy UV lamp to ionize chemicals in the gasses for producing the detection signal, provide the advantage of excellent sensitivity and simplicity, but the main limitation is that measurements are not compound-specific. Also, electrochemical gas sensors work by allowing the gas to diffuse through a porous membrane to an electrode, hence producing an amount of electric current utilized for examining the concentration of the gas. Since the diffusion barrier of electrochemical sensors is a physical barrier, it tends to be more reliable and stable over the sensor’s duration. Nevertheless, the electrochemical sensors are subject to chemical contamination or corrosive elements and may last only 1–2 years before a replacement is required. Concerning the QD gas sensor in our model, owing to their working principles similar to the MOS sensors, this sensor type works over a smaller distance than particular other sensor types. In our simulation, we obtain that charge transfer between the neighboring QDs similar to the hopping mechanism in photosynthesis. However, QD gas sensors may be disadvantaged by the cross-sensitivity with humidity, but such a problem can be improved by a certain baseline correction mode, as demonstrated in the study by Ghosh et al. (2019).

## 5 Conclusion

In this study, we apply the master equation to analyze the electron transport in the QD gas sensor for a wide range of parameters. Effects of the perturbed Schottky (*λ*_*j*,*j*+1_) and the temperature (Γ_0_) are also considered.

Our analysis shows that the temperature and the two different gas types affect the signal of the QD gas sensors. The higher temperature plays a role in accelerating electron transport, increasing the energy current and ETE, and shortening the time duration of the transport.

The gas detection effects perturb the Schottky barrier, increasing or decreasing the depletion layer and height of the potential between QDs, corresponding with adsorption oxidizing and reducing gasses, respectively. The oxidizing gasses act as electron acceptors upon adsorption on the surface of QDs as n-type semiconductor materials, while the reducing gasses act as electron donors. The former reduces the electrical conductance of the structure, and the latter leads to an increase in the conductance. For this reason, the oxidizing and reducing gasses cause different changes in the electrical conductance. In our model, this mechanism is represented by the value of *λ*_*j*,*j*+1_ parameter. The decreased depletion layer and height of the potential (high value of *λ*_*j*,*j*+1_) make electron transfer between QDs more comfortable, while the increased depletion layer and height of the potential (low of *λ*_*j*,*j*+1_) hinder the electron transfer. According to our model, the electron transport characteristic in the QD gas sensor resembles the energy transfer in plants’ photosynthesis. In QD gas sensors, the electron is transferred from the initial QD to the final QD with the involvement of the temperature modeled by phonon heat baths. The energy transfer of plants’ photosynthesis, in the same manner, transports from the initial chlorophyll’s pigment to the final pigment named reaction center. In the photosynthesis process, the energy transport corresponds to the phonon baths of each pigment depending on the temperature, similar to the QD gas sensor’s energy transport in which the electron is a carrier between QDs. Moreover, the results in our work concerning the QD gas sensor entail the coupling energy *λ*_*j*,*j*+1_ between QDs in common with the hopping energy between the pigments in the photosynthesis process.

Compared to other gas sensor types, the QD gas sensor, like most MOS sensors, works over a smaller distance, but may be disadvantaged by the cross-sensitivity with humidity that can be amended. Based on our theoretical model, to manufacture QD gas sensors, temperature control is a crucial factor. In addition, the response of the sensors should involve the detection of the energy current’s alteration in QDs by the effects of the perturbed Schottky barrier from the gasses. We hope that our work might provide helpful insights into the electron transfer mechanisms in QD gas sensors and will be constructive for designing novel nanofabricated gas sensing.

## Data Availability

The original contributions presented in the study are included in the article/Supplementary Material; further inquiries can be directed to the corresponding author..
